# A novel digital arthrometer to measure anterior tibial translation

**DOI:** 10.1186/s13018-022-03497-4

**Published:** 2023-02-13

**Authors:** Danni Wu, Donghai Wang, Yongjie Han, Luqi Guo, Shaobai Wang

**Affiliations:** 1 Key Laboratory of Exercise and Health Sciences of Ministry of Education, Shanghai University of Sport, 200 Hengren Road, Yangpu District, Shanghai, China; 2 Department of Orthopedics, The First Affiliated Hospital of Soochow University, Orthopedic Institute of Soochow University, 899 Pinghai Road, Soochow, China; 3 The Second Affiliated Hospital of Inner Mongolia Medical University, Orthopedic Institute of Inner Mongolia Autonomous Region, 59 Horqin South Road, Saihan District, Hohhot, China; 4 Laboratory of Biomechanics and Engineering, Innomotion, 518 Xinzhuan Road, Caohejing Development Zone, Shanghai, China

**Keywords:** Knee laxity, Anterior tibial translation, Digital arthrometer, Anterior cruciate ligament, Quantitative study

## Abstract

**Background:**

Measurement of knee laxity after anterior cruciate ligament (ACL) injury is crucial for appropriate treatment and rehabilitation decision-making. This study examined the potential of a new digital arthrometer (Ligs, Innomotion, Shanghai, China) to quantify anterior tibial translation (ATT) in patients with ACL injuries and in healthy subjects.

**Methods:**

A total of 60 participants included 30 subjects with single-leg ACL injuries and 30 healthy subjects included as controls. The lower leg was immobilized. The thruster is positioned posterior to the lower leg and parallel to the tibial tuberosity in the sagittal plane. The load is applied vertically to the tibia under a dynamic load of 0–150 N, with continuous displacement recorded. The intrarater and interrater reliability will be examined. ATT and side-to-side differences (SSD) between the control and ACL injury groups were compared. Receiver operating characteristic (ROC) curves were analyzed, and the area under the curve (AUC) was calculated to determine the diagnostic accuracy of the Ligs.

**Results:**

The interrater ICC was 0.909 and the intrarater ICC was 0.943. Significant differences in the SSD were observed between the control and ACL injury groups (for all *P* < 0.05), with the largest effect size (ES = 1.12) at 80 N. When comparing ATT at different loads between injured and healthy sides in the ACL injury group, displacement was statistically significant at different loads. At a load of 150 N, the AUC was the maximum (0.857) and the sensitivity and specificity were 0.87 and 0.73, respectively.

**Conclusions:**

A digital arthrometer can be used as a quantitative instrument to quantify knee laxity. Quantitative measurement of ATT and SSD under controlled loading can be an objective and effective tool for clinical practice.

## Background

The basic function of the anterior cruciate ligament (ACL) is to limit excessive anterior displacement and valgus rotation of the tibia relative to that of the femur. ACL injuries are frequently observed [11]. Furthermore, the evaluation of knee laxity after ACL injury is important in deciding the treatment plan. Magnussen et al. [17] found that individuals with greater preoperative knee laxity were significantly more likely to undergo ACL revision surgery in the following 6 years. Additionally, knee laxity is associated with a high risk of ACL reconstruction failure [1]. In a study evaluating factors influencing meniscal injury during ACL reconstruction, Nakamae et al. [19] found that the high incidence of meniscal injuries was closely related to the high laxity of the knee joint and was more prevalent in men. Therefore, it is of great clinical importance to develop a simple method that can quantitatively and accurately assess knee laxity after ACL injury.

Physical examination is commonly used to assess knee laxity, although the condition cannot be quantified by physical examination alone. Recently, several devices have been introduced to quantify anterior tibial translation (ATT) that can be widely used to objectively assess knee laxity [21, 22, 24]. For example, the KT1000 (Medmetric Corp, San Diego, CA, USA) device has also been used extensively to assess knee laxity after ACL injury [23]. Wiertsema et al. [28] compared the reliability of the KT1000 device with that of the Lachmann test in individuals with ACL rupture. The reliability of the KT1000 was lower than that of the Lachman test, with intraclass correlation coefficient (ICC) for both intrarater (1.0 vs 0.47) and interrater (0.77 vs 0.14) reliability and repeated measurements of the KT1000 arthrometer in a single measurement session showing inadequate reliability. Runer et al. tested the inter- and intrarater reliability of four different knee arthrometers (KLT, Karl Storz,KiRA, I + ; KT-1000, Medmetric Corp; Rolimeter, Aircast) in healthy patients and obtained ICC ranging from 0.49**–**0.70 [25]. Thus, easy-to-use and accurate instruments to quantify knee laxity are currently not available.

**Fig. 1:**
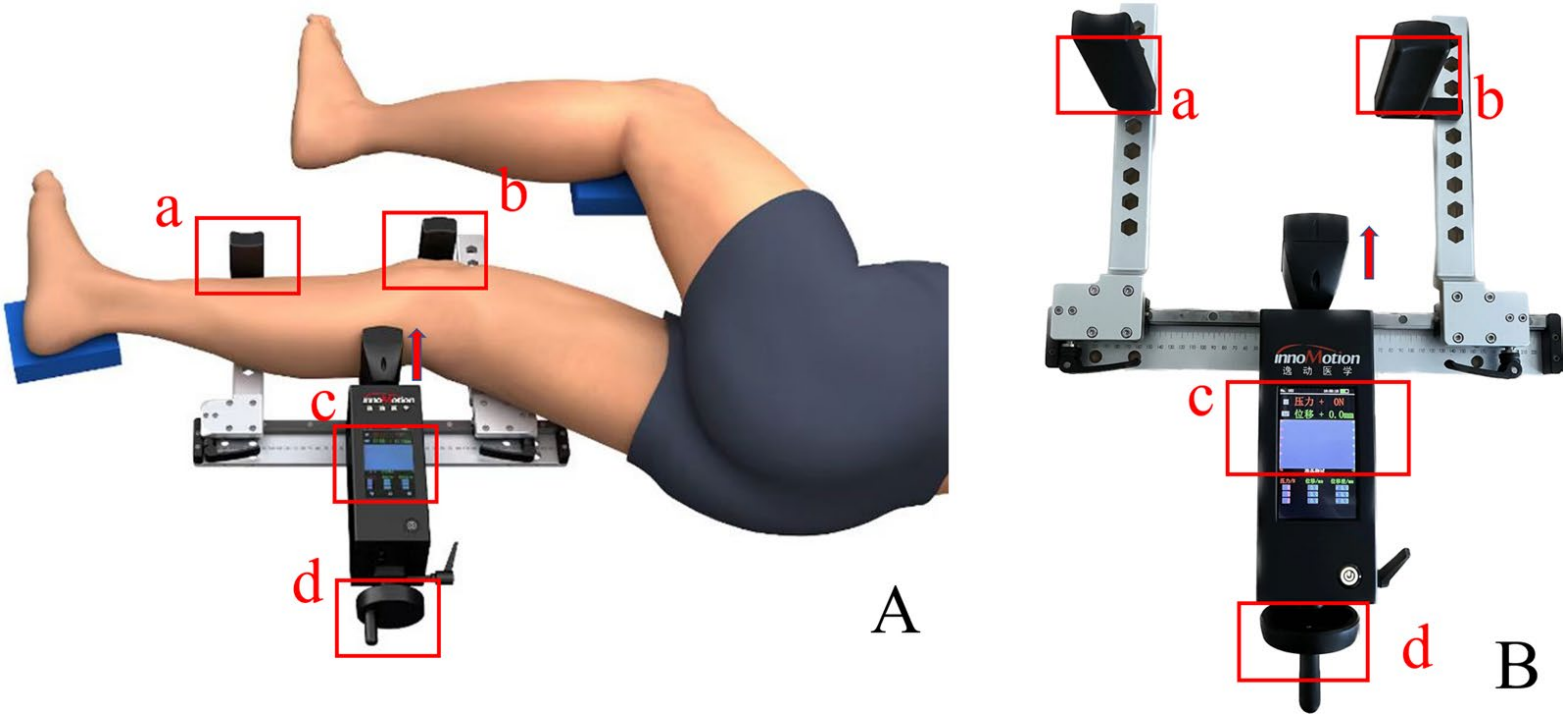
**A** Measuring anterior tibial translation (ATT) using the Ligs device. **B** Illustration showing Ligs components. a: shape I component. b: shape L component. c: mainframe display screen (data acquisition interface). The first line of the screen represents the instantaneous load (N). The second line represents the instantaneous displacement (mm). The three columns of measured values at the bottom of the interface, from left to right, respectively, represent loads, corresponding displacements and side-to-side differences. User can customize three typical loads to observe the corresponding displacements and side-to-side differences. d: thruster. The arrow indicates the direction of the applied load

Recently, a new digital arthrometer (Ligs, Innomotion, Shanghai, China) was developed to optimize the quantification of knee laxity in terms of its size and operation. It is similar in appearance to the Telos device. However, Ligs uses a digital intelligent sensor to acquire ATT, replacing the measurement on the X-ray image. It has the advantage of avoiding radiation exposure while simplifying operation. The Ligs is a portable, non-invasive device for joint laxity testing consisting of a main frame and a fixing bracket. The frame has built-in load and displacement sensors at a sampling rate of 30 Hz that continuously record the real-time load and displacement. The accuracy of the load is 1 N, and the accuracy of the displacement is 0.1 mm. The limit bracket is primarily used to fix the body position (Fig. 1B). Chen et al. used the Ligs device to quantify anterior drawer testing (ADT) in chronic ankle instability with satisfactory results [6]. However, the feasibility of quantifying ATT using this device has not yet been analyzed.

Therefore, the purpose of this study was to investigate whether the Ligs device has the potential to quantify knee laxity. Since patients with ACL injuries have significantly higher ATT than healthy individuals, ATT was compared in healthy and ACL-injured knees to determine differences in quantification parameters, and intra- and interrater reliability. Our hypothesis was that the Ligs device would be able to objectively quantify knee laxity.

## Methods

The study was conducted in accordance with the Declaration of Helsinki and was approved by the Ethics Committee (No. 102772021RT040 of our institution). All participants signed an informed consent form prior to the start of the study.

### Participants

A total of 60 participants were included: 30 participants with unilateral ACL injury and 30 healthy volunteers.

Participants in the ACL injury group were diagnosed with isolated and complete ACL rupture by a sports medicine specialist based on clinical symptoms and magnetic resonance imaging results. The inclusion criteria were as follows: (i) single-leg ACL injury, (ii) complete ACL rupture, (iii) preparation for ACL reconstruction surgery after examination, (iv) Lachman test ( +), and (v) body mass index (BMI) < 30. The exclusion criteria were (i) age < 18 or > 45 years, (ii) ACL injury in both knees, (iii) limitation of knee motion (knee flexion limitation), (iv) chronic ACL injury (> 3 months after injury), (v) BMI ≥ 30, (vi) previous history of knee pain or knee surgery, (vii) injury to the muscles, tendons, ligaments or cartilage around the knee (except ACL), (viii) degenerative knee disease.

Participants in the control group satisfied the following conditions: (i) no previous history of knee pain or knee surgery, (ii) no injury to the muscles, tendons, ligaments or cartilage around the knee, (iii) no degenerative knee disease, and (iv) BMI < 30.

### Experimental process

All subjects were required to be free of strenuous activity for 48 h prior to examination and wore black shorts during testing. Subjects were placed in a lateral position with the bilateral lower limbs exposed and the knees flexed at 30° simulating the Lachman test position. The components on one side of the fixator were placed anteriorly near the patella to limit femoral motion, and on the other side, distal to the tibia. Subsequently, a pushing force was gradually applied from the posterior of the tibia. The thruster at the end of the main frame were set at a constant speed (3 N/s) to apply a thrust from the back of the lower leg, causing anterior displacement of the tibia (Fig. 1A). To mitigate the effects of muscle tissue, the displacement was recorded when the pressure exceeded 20 N and an alarm was sounded when the force reached the set value. In this study, the upper load threshold was set at 150 N. In a previous study, Bercovy et al. [5] found that the minimum load for an accurate diagnosis of ACL rupture was 180 N. In our pre-test findings, three control subjects were unable to reach this threshold. Furthermore, the KT1000 selected 134 N as the maximum threshold for examination. Therefore, 150 N was selected as the maximum threshold. Displacements corresponding to fixed loads of 80 N, 120 N, and 150 N were recorded. The side-to-side differences in the control group were calculated by subtracting the ATT of the non-dominant side from the dominant side. The side-to-side differences in the ACL injury group were calculated by subtracting the ATT of the healthy side from the injured side. The interrater reliability was tested for two independent examiners using the same group of ten healthy subjects. The intrarater reliability was tested on the same subjects by the same examiner at three different time points (1-h intervals between the three tests). Both inter- and intrarater reliability were tested by measuring ATT values using the Ligs device. The technical dimensions of ATT and SSD are measured in millimeters (mm). The accuracy of load was 1 N and the displacement was 0.1 mm. All measurements were conducted three times, and the average value was recorded.

### Statistical analysis

Statistical analysis was performed using IBM SPSS software (version 23.0, Armonk, NY, USA). The ICC was calculated to test the intra- and interrater reliability. An ICC greater than 0.74 was considered excellent, an ICC between 0.60 and 0.74 was considered good, an ICC between 0.40 and 0.59 was considered fair, and values less than 0.40 were considered poor as in a previous study [7].

The Kolmogorov–Smirnov test was used to verify whether the following continuous variables conformed to a normal distribution: age, BMI, ATT, and side-to-side differences (SSD). A chi-square test was used to compare the gender differences between the control and ACL injury groups. Independent samples t test was used to compare the differences in age and BMI between the control and ACL injury groups. An independent sample t test was used to compare the control and ACL injury groups with the SSD under loads of 80 N, 120 N, and 150 N. A paired sample t test was used to compare the mean ATT of the ACL injury group on the healthy and injured sides under loads of 80 N, 120 N, and 150 N. Effect sizes were calculated and compared. Cohen's d effect size classification was defined 0.2 as small, 0.5 as medium, and 0.8 as a large effect [8]. Receiver operating characteristic (ROC) curve analysis was used to calculate the area under the curve (AUC) and to determine cutoff values for the control and ACL injury groups. Sensitivity and specificity were also calculated. The significance level was set at *P* < 0.05.

## Results

### Demographics

The characteristics of the subjects were compared between the control and ACL injury groups (Table 1). There were no statistically significant differences with regard to sex (*P* = 0.176), with the control group being 56% males and the ACL injury group being 73% of males. There were no statistically significant differences in age between the groups (*P* = 0.198). In the ACL injury group, the right side was involved more often, accounting for approximately 67% of the total number of injuries. There were no statistically significant differences in BMI (*P* = 0.791).

**Table 1:** Subject demographic characteristics^a^

Variable	ACL injury group (*n* = 30)	Control group (*n* = 30)	*P* value
Gender(M/F)	22/8	17/13	0.176
Age(Y)	23 ± 3.3	22 ± 3.0	0.198
Injured side (L/R)	13/17	–	–
BMI (kg/m^2^)	21.1 ± 2.1	20.9 ± 2.3	0.791

*ACL* anterior cruciate ligament, *BMI* body mass index, *M* Male, *F* Female, *Y* Year, *L* Left, *R* Right

^a^Values are presented as mean ± standard deviation (SD); the level of significance was established a priori at *P* < 0.05

### Inter- and intrarater reliability assessment

The interrater reliability was considered excellent with an ICC score of 0.909 (95% CI, 0.853–0.945) and a mean measured value of 0.953 (95% CI, 0.921–0.972). The intrarater reliability was considered excellent with an ICC score of 0.943 (95% CI, 0.918–0.961) for a single metric and a mean measure of 0.971 (95% CI, 0.957–0.980).

### Reference standard comparisons

Comparison of SSD under different loads in the control and ACL injury groups showed that SSD was significantly greater in ACL-injured subjects at 80 N, 120 N, and 150 N (all *P* < 0.01). The largest effect size was observed at a load of 80 N (effect size = 1.12, Table 2).

**Table 2:** Comparison of SSD in the ACL injury group and the control group^a^

Variable	SSD of the ACL injury group (mm)	SSD of control group (mm)	*P* value	95%CI	Effect size
80 N	2.7 ± 1.4	1.2 ± 1.1	0.000***	0.77–2.07	1.12
120 N	3.4 ± 2.2	1.7 ± 1.2	0.001**	0.74–2.59	0.93
150 N	3.8 ± 2.7	1.8 ± 1.3	0.001**	0.92–3.17	0.94

*SSD* side-to-side difference, *ACL* anterior cruciate ligament, *CI* confidence interval

^a^Values are presented as mean ± standard deviation (SD); the level of significance was established a priori at *P* < 0.05

^***^P < 0.001, **P < 0.01

**Fig. 2:**
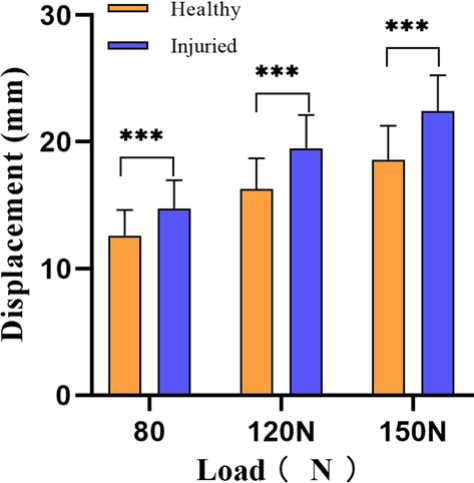
ATT at different loads between the injured and healthy sides of the ACL injury group. Values are presented as mean ± standard deviation (SD), ***P < 0.001. ATT: anterior tibial translation

**Fig. 3:**
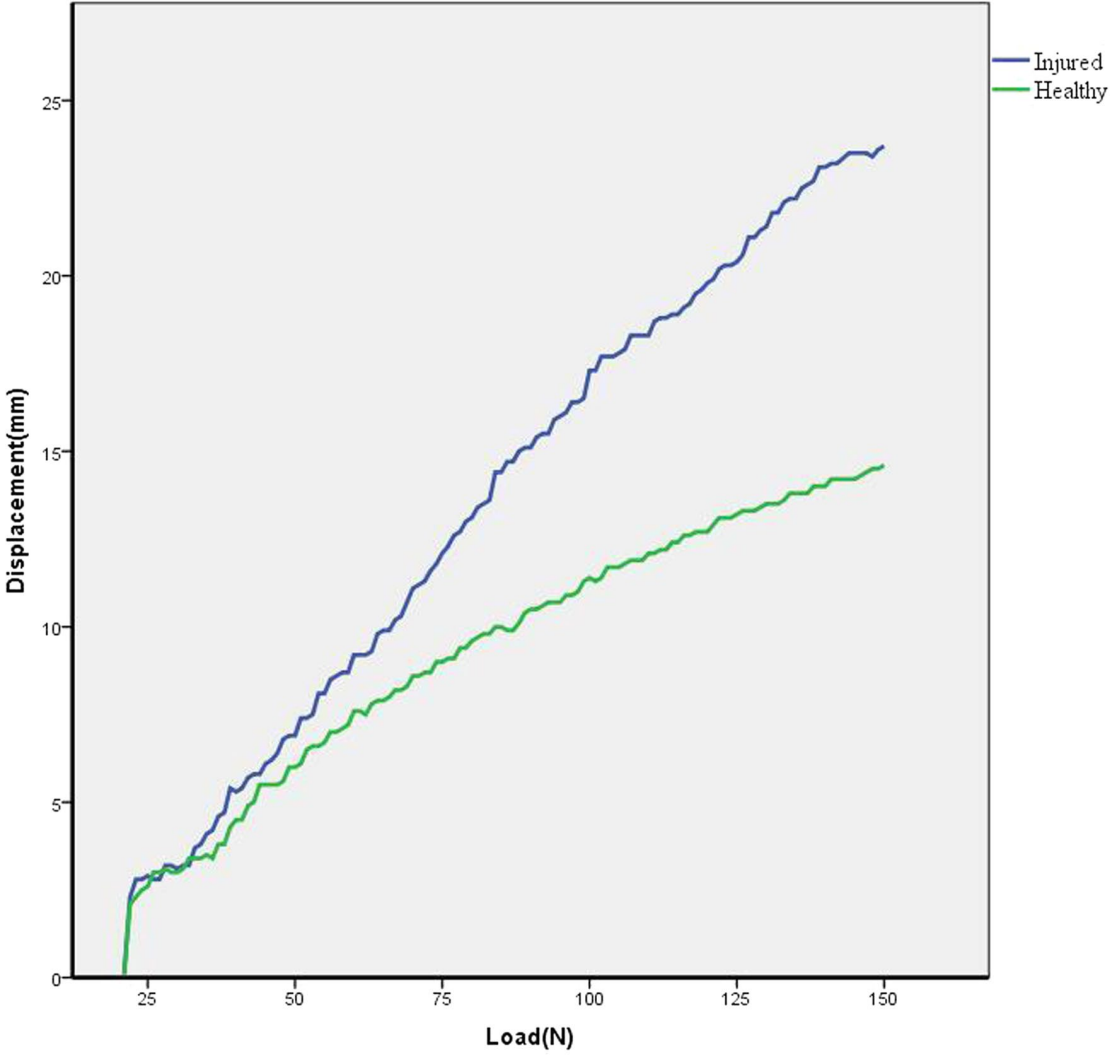
Typical load–displacement curves for the healthy and injured sides in the ACL injury group. The green is selected from the healthy side and the blue is selected from the injured side. A steeper curve is observed on the injured side with greater laxity compared to the healthy side. *ACL* anterior cruciate ligament

When comparing ATT at different loads between injured and healthy sides in the ACL injury group, displacement was significantly greater in ACL-injured subjects at 80 N, 120 N, and 150 N (all *P* < 0.001) (Fig. 2). The largest effect size was observed at a load of 150 N (effect size = 1.40, Table 3). The curves indicating typical load–displacement changes in healthy and injured knees are shown in Fig. 3. Compared with the healthy side, the curve on the injured side was steeper, indicating greater laxity.

**Table 3:** Comparison of ATT on injured and on healthy sides in the ACL injury group^a^

Variable	ATT of injured side (mm)	ATT of healthy side (mm)	*P* value	95%CI	Effect size
80 N	14.7 ± 2.2	12.6 ± 2.0	0.000***	1.37–2.95	1.02
120 N	19.5 ± 2.6	16.3 ± 2.4	0.000***	2.34–4.12	1.28
150 N	22.4 ± 2.8	18.6 ± 2.7	0.000***	2.83–4.86	1.40

*ATT* anterior tibial translation, *ACL* anterior cruciate ligament, *CI* confidence interval

^a^Values are presented as mean ± standard deviation (SD); the level of significance was established a priori at *P* < 0.05

^***^P < 0.001

### Diagnostic accuracy

**Fig. 4:**
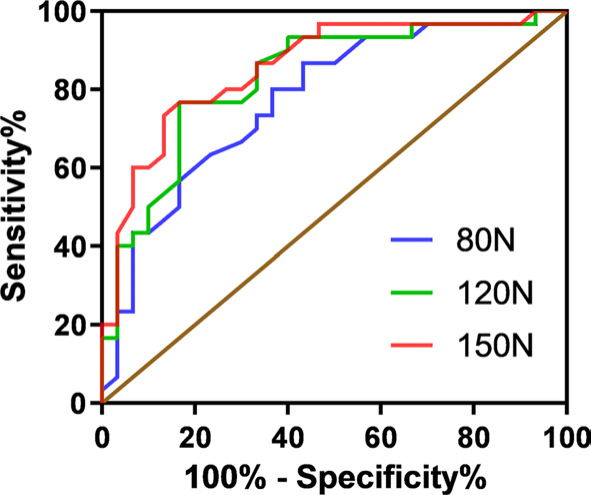
ROC curves of ATT under different loads. *ACL* anterior cruciate ligament, *ROC* receiver operating characteristic

A load of 150 N produced the maximum AUC (0.857, 95%CI [0.761–0.954]), the cutoff value was set to 19.7 mm, and the sensitivity and specificity were 0.87 and 0.73, respectively. The ROC curves for the different loads are shown in Fig. 4.

## Discussion

In this study, the results showed excellent inter- and intrarater reliability (ICC scores of 0.909 and 0.943, > 0.75, defined as excellent) for measuring ATT values using the Ligs device, which indicates its suitability for the quantification of knee laxity. Meanwhile, ATT and SSD under different loads were significantly greater in ACL injury group than that in the control group. Our study confirmed our hypothesis that digital arthrometry could be used as a tool for the quantitative assessment of knee laxity.

Knee laxity after ACL injury can be compensated by muscle strengthening and neuromuscular proprioceptive exercises [9]. Increasing knee laxity may be an early signal of knee decompensation and structural damage when compensatory mechanisms have been triggered [16]. Therefore, a simple and objective approach to following up patients with ACL injuries is by evaluating knee laxity.

The Lachman test (30° stress physical examination) is a screening tool commonly used by clinicians with high sensitivity (94%) and specificity (83%) [18]. However, the results of the Lachman test can be influenced by the clinical experience of the examiner, as well as their subjective perceptions [14]. Further, the Lachman test does not provide any quantitative indicators. The clinician grip configuration influenced the performance and interpretation of the Lachman test [29].

The KT1000 arthrometer is the most common instrument for measuring ATT and has been reported to be suitable for the diagnosis of ACL injuries because of its high sensitivity (92%) and specificity (95%) [2–4, 12, 27]. However, Forster et al. [10] found significant inter- and intrarater variability (ICC = 0.14 and 0.47) in measurements of ATT and SSD using the KT1000. In the study by Sernert et al. [26], the KT-1000 was used to analyze and compare knee laxity between left- and right-handed dominant physical therapists in patients with ACL injuries. Left hand-dominant therapists obtained significantly higher values for left knee laxity. The device we used applied the load uniformly (3 N/s) through a hand crank at the end of the mainframe (Fig. 1B). The test results were not affected by the dominant hand. The Telos device (GmbH, Hungen, Germany) is widely used as a mobile stress stent in conjunction with X-rays to diagnose ACL injuries and showed a sensitivity of 86.0% and a specificity of 89.2% at 30° for knee flexion using 3 mm as the threshold value [15]. However, radiation exposure is its main drawback. The Ligs is a portable, non-invasive, nonradiation-dependent, quantitative examination device with excellent reliability.

The Ligs device quantifies ATT using built-in sensors, recording loads and displacements in real time. Our study showed ICC of 0.909 and 0.943 for inter- and intrarater, respectively, confirming the reliability of applying Ligs to detect knee laxity after ACL injury. Furthermore, the results of the Ligs test are quantitative indicators that can reduce experiential dependence. The Ligs device was also used by Chen et al. for the assessment of chronic ankle instability and was found to have excellent intra- and interrater reliability with ICCs of 0.963 and 0.949, respectively [6]. Our study focused on testing knee laxity, a component whose potential has not been reported. This is one of the novelties of our study.

In our study, the mean SSD for the ACL injury group was 2.7 mm at 80 N, with a maximum AUC of 0.782 (95% CI, 0.666–0.898) and an effect size of 1.12. The results indicated that the presence of ACL injuries could be identified using SSD at lower loads. We recommend the use of 80 N as the optimal load to quantify the diagnosis of ACL injury to prevent discomfort in the patient. A larger SSD decreases the sensitivity of the diagnosis; however, its specificity is significantly higher, effectively reducing false-positive cases. The mean SSD of healthy participants in the control group was 1.2 mm. Niu et al. [20] used an automatic knee arthrometer to measure knee laxity after ACL injury and found that SSD was less than 1.5 mm in healthy participants. We infer that SSD in healthy individuals is less than 1.5 mm.

In the ACL injury group, the ATT of the healthy and injured sides at different loads were statistically significant (*P* < 0.05). With increasing load, the mean ATT value at 150 N had a maximum AUC of 0.857. The cutoff value was set at 19.7 mm with sensitivities and specificities of 0.87 and 0.73, respectively. A large effect size was observed at a load of 150 N (effect size = 1.40). This result suggests that at a load of 150 N, the ATT exceeding 19.7 mm may be a sign of an ACL injury. Furthermore, Keizer and Otten [13] in their review concluded that the mean ATT was lower in controls (5.96 mm) and in the healthy contralateral side (5.33 mm) than in the ACL-injured knees (9.15 mm), which was also confirmed by our findings.

One of the limitations of the study is the effect of soft tissue deformation. To mitigate the effect of muscle tissue, the displacement was recorded when the pressure exceeded 20 N. We compared the ATT bilaterally and calculated the SSD. The left and right legs of the same subject had similar soft tissue deformation, and bilateral comparisons further reduced the effect of soft tissue deformation. The increment of displacement produced by soft tissue deformation had less effect on the results. In addition, we attempted to maintain the thruster to apply the load in the sagittal plane by ensuring several requirements were met. Firstly, the subject’s body position was strictly controlled. The subject was placed in the lateral position with the examined leg close to the examination bed and the knee joint flexed at 30°. The distal end of the tibia was elevated with a pad to maintain horizontal placement of the tibia. The other leg was flexed and placed in front of the body so that the body was naturally relaxed. Secondly, the anatomical landmarks of the fixation position were clarified. The thruster was positioned posterior to the lower leg and parallel to the tibial tuberosity in the sagittal plane. Finally, the load was applied vertically forward on the tibia. In future studies, we suggest further estimation of soft tissue deformation to improve measurement accuracy. In our study, we did not measure the translation of the ventral tibia relative to the femur. The measurement method is similar to that of Telos equipment. This makes the data more comparable. In future studies, it should be further observed whether the tibial translation measured ventrally is the same as that measured dorsally.

Furthermore, the participants were limited to patients with simple ACL injuries, and thus, further in-depth studies should be conducted to include patients with different types of ACL injuries and to differentiate population characteristics. More accurate diagnostic criteria can be refined by including different subject populations to obtain enriched ATT data.

## Conclusions

A digital arthrometer can be used as a quantitative instrument to quantify knee laxity. Quantitative measurement of ATT and SSD under controlled loading can be an objective and effective tool applicable to clinical practice, and ACL injuries can be identified by SSD at lower loads. The highest diagnostic accuracy of SSD under an 80 N load and ATT under a 150 N load can provide an objective scientific basis to aid in the diagnosis of ACL injury in the clinical setting. A practical comparison with the established devices is necessary in future.

## Data Availability

The datasets generated during and/or analyzed during the current study are available from the corresponding author on reasonable request.

## Abbreviations

ACL: Anterior cruciate ligament
ATT: Anterior tibial translation
SSD: Side-to-side differences
ICC: Intraclass correlation coefficient
ROC: Receiver operating characteristic
AUC: Area under the curve
ADT: Anterior drawer testing
BMI: Body mass index
SD: Standard deviation
CI: Confidence interval
